# Supplementary material for: Mental health and suicide risk among homosexual males in Bangladesh
Source: PLoS One. 2023 Aug 15;18(8):e0289597. doi: 10.1371/journal.pone.0289597 (PMC10426927; doi:10.1371/journal.pone.0289597)
Supplement: Attachment — Submitted filename: Response to Reviewers.docx [file pone.0289597.s003.docx]

**1. Response to Reviewer.**

Summary of research and reviewer comment

The author has raised a very important issue in the paper and the overall impression of research paper is good. Gender diversity and sexual orientation are complex and often taboo topics in Bangladesh. The author has shown some audacity while deciding investigating this sensitive yet critical social phenomenon.

***Response:*** Thank you for agreeing to review this article and making these comments to improve the quality of the article.

Minor discussion

-While the author talk’s suicide cases among homosexual, I really think the broader scenario of issue need to be discussed in introduction and literature review. For instance its prevalence in developed world, than developing and under developed world. In addition to that while you talk of Bangladesh, it is important for international reader to give an overall view of your country. A little literature on country’s geographical location, socio economic indicators and population dynamics will help understanding the presence of certain social phenomenon in country.

***Response:*** Thank you for these suggestions. We have included a brief summary of contextual details on Bangladesh in the introduction. However, we did not include a comparative analysis of suicide rates in developed, developing and underdeveloped worlds. Our initial planning had that in mind, but as there are many factors associated with the rates of suicide in a specific context, we felt it would require a carefully planned meta-analysis to make a meaningful comparison in that regard. We, therefore, avoided such premature misleading comparisons, which we could achieve through a brief literature review.

*Added section:*

Bangladesh is a densely populated (1140/square km) south Asian country with a population of 169.1 million [1]. The majority of the population is Muslim (88.4%) and aged between 15-49 years (54.9%), with a male-to-female ratio of 100.2 [1]. Male homosexuals are a hidden sexual minority group in Bangladesh, with a decade-old estimation of the homosexual population indicating a size between 21833 to 110581 [2].

-The parental role and investigation from respondents about the way the respondents have been raised by parent’s was a big missing part in research.

***Response:*** Yes, that would add a wider perspective in interpreting the data; however, we did not collect data on the parental role. As the data collection is already completed, we may only try to accommodate that in future research.

-Similary, It would have been great if reaction was investigated from respondents about their thoughts and parents own perception while knowing their children are homosexual and that effect on their mental health.

***Response:*** This is another area that we cannot address now, as the data collection is already completed, we will try to accommodate this in future research.

-A literature could also cover the legality of such sensitive issue in Bangladesh as an Islamic state particularly or anything in pipeline by state while addressing homosexuality in country.

***Response:*** We have revised the writing and added a sentence to address this.

*Revised and added section:*

Bangladesh being a Muslim-majority country, the disapproval of homosexual behavior is rooted in religious scriptures [Jami at-Tirmidhi, hadith 1456; Sahih Al-Bukhari, hadith 6834] and supported by the national penal codes. Keeping the British colonial track, Bangladesh penal code criminalizes any voluntary carnal intercourse between men and termed it as “against the order of nature” [Section 377; 6]. Despite the legal position, the government of Bangladesh is supporting activities towards improving the health and psychosocial care of homosexuals and other sexual minority groups, especially in connection to HIV/AIDS prevention program [see 7]

-The author can avoid references that are too old, like one mentions year 1984 and so.

***Response:*** Thank you for this suggestion. We have checked the document thoroughly and removed a few old citations while adding a few new citations. Subsequent changes in reference sections have also been made.

Removed:

Herek (1984); Stein and Cohen (1984); Kourany, (1987); Robinson and Price (1882); Beck, et al (1990); Beck and Steer (1988)

Added:

Bangladesh Bureau of Statistics, (2021); icddrb and Government of Bangladesh, (2012); Margolin, (2023); National AIDS/STD Programme (NASP) (2016); Kanbur, (2020); Taghavi, (2002); Liu et al., (2020); Baryshnikov et al., (2020);

**2. Response to Academic Editor.**

1. Please ensure that your manuscript meets PLOS ONE's style requirements, including those for file naming. The PLOS ONE style templates can be found at

***Response:*** Thank you for this suggestion, we had missed a few in the last submission. Now we have revised the manuscript as per PLOS ONE style requirements.

2. Please include a complete copy of PLOS’ questionnaire on inclusivity in global research in your revised manuscript. Our policy for research in this area aims to improve transparency in the reporting of research performed outside of researchers’ own country or community. The policy applies to researchers who have travelled to a different country to conduct research, research with Indigenous populations or their lands, and research on cultural artefacts. The questionnaire can also be requested at the journal’s discretion for any other submissions, even if these conditions are not met.  Please find more information on the policy and a link to download a blank copy of the questionnaire here: https://journals.plos.org/plosone/s/best-practices-in-research-reporting. Please upload a completed version of your questionnaire as Supporting Information when you resubmit your manuscript.

***Response:*** We have completed the questionnaire and attached that as supporting information.

3. In the ethics statement in the Methods, you have specified that verbal consent was obtained. Please provide additional details regarding how this consent was documented and witnessed, and state whether this was approved by the IRB

***Response:*** We have revised the secion as per the suggestion

*Revised and added section:*

Two-stage verbal consent process was used in selecting participants. In the first step, the NGO staff asked the prospective participants if they were interested in joining this study. Based on their approval, the staff sent the prospective participants to the interviewers, where final consent was sought. The interviewer informed the participants about the purpose, procedures, confidentiality, and voluntary nature of the study. Verbal informed consent was taken instead of written consent to protect the participants’ identity [42] and to avoid legal ramifications [as homosexuality is prohibited by law; 6] in this sensitive research topic. This research was approved by the research Ethics Committee of the Department of Clinical Psychology, University of Dhaka (project number: RR-221101).

4. Please include your full ethics statement in the ‘Methods’ section of your manuscript file. In your statement, please include the full name of the IRB or ethics committee who approved or waived your study, as well as whether or not you obtained informed written or verbal consent. If consent was waived for your study, please include this information in your statement as well.

***Response:*** We have revised the statement as per the suggestion

*Revised IRB statement:*

This research was approved by the research Ethics Committee of the Department of Clinical Psychology, University of Dhaka (project number: RR-221101).

5. Please review your reference list to ensure that it is complete and correct. If you have cited papers that have been retracted, please include the rationale for doing so in the manuscript text, or remove these references and replace them with relevant current references. Any changes to the reference list should be mentioned in the rebuttal letter that accompanies your revised manuscript. If you need to cite a retracted article, indicate the article’s retracted status in the References list and also include a citation and full reference for the retraction notice.

***Response:*** In line with the suggestions from the reviewer, we have revised the reference section, where 6 very old references were removed, and 8 references were added.

| Removed:  Herek (1984);  Stein and Cohen (1984);  Kourany, (1987);  Robinson and Price (1882);  Beck, et al (1990);  Beck and Steer (1988) | Added:  Bangladesh Bureau of Statistics, (2021);  icddrb and Government of Bangladesh, (2012);  Margolin, (2023);  National AIDS/STD Programme (NASP) (2016);  Kanbur, (2020);  Taghavi, (2002);  Liu et al., (2020);  Baryshnikov et al., (2020); |
| --- | --- |
