# Supplementary material for: Mental health and suicide risk among homosexual males in Bangladesh
Source: PLoS One. 2023 Aug 15;18(8):e0289597. doi: 10.1371/journal.pone.0289597 (PMC10426927; doi:10.1371/journal.pone.0289597)
Supplement: Attachment — Submitted filename: Summery of research and reviewer comment.docx [file pone.0289597.s002.docx]

Summary of research and reviewer comment

The author has raised a very important issue in the paper and the overall impression of research paper is good. Gender diversity and sexual orientation are complex and often taboo topics in Bangladesh. The author has shown some audacity while deciding investigating this sensitive yet critical social phenomenon.

Minor discussion

-While the author talk’s suicide cases among homosexual, I really think the broader scenario of issue need to be discussed in introduction and literature review. For instance its prevalence in developed world, than developing and under developed world. In addition to that while you talk of Bangladesh, it is important for international reader to give an overall view of your country. A little literature on country’s geographical location, socio economic indicators and population dynamics will help understanding the presence of certain social phenomenon in country.

-The parental role and investigation from respondents about the way the respondents have been raised by parent’s was a big missing part in research.

-Similary, It would have been great if reaction was investigated from respondents about their thoughts and parents own perception while knowing their children are homosexual and that effect on their mental health.

-A literature could also cover the legality of such sensitive issue in Bangladesh as an Islamic state particularly or anything in pipeline by state while addressing homosexuality in country.

-The author can avoid references that are too old, like one mentions year 1984 and so.
